# Making Plant Physiology Visible: Ten Inquiry-Based Infrared Thermography Activities for Undergraduate Teaching

**DOI:** 10.3390/plants15213446

**Published:** 2026-11-05

**Authors:** Carlos Frey, José Luis Acebes

**Affiliations:** 1 Área de Fisiología Vegetal, Departamento de Ingeniería y Ciencias Agrarias, Facultad de Ciencias Biológicas y Ambientales, Universidad de León, Campus Vegazana, 24007 León, Spain; 2 Centro de Biotecnología y Genómica de Plantas (CBGP), Universidad Politécnica de Madrid, Campus de Montegancedo, Ctra, M-40, km 38, 28223 Madrid, Spain

**Keywords:** active learning, infrared thermography, inquiry-based learning, plant physiology, plant science education, thermal imaging, undergraduate laboratory

## Abstract

Many of the processes studied in plant physiology, including stomatal regulation, responses to drought, formation of graft unions, interactions with pathogens, floral thermogenesis and freezing, are dynamic but difficult for students to observe directly. Consequently, teaching these topics often relies on indirect measurements or theoretical explanations, which makes it difficult for students to understand the link between physiological concepts and observable plant behaviour. Infrared thermography offers a rapid, non-invasive way to visualise temperature patterns that reflect underlying physiological processes. This article presents ten inquiry-based laboratory and field activities that integrate infrared thermography into undergraduate plant physiology teaching. Rather than focusing on the technology itself, the activities are organised around fundamental physiological concepts, challenging students to formulate hypotheses, interpret thermal images and relate temperature patterns to plant function. The activities cover topics such as water relations and transpiration, graft union formation, plant–pathogen interactions, drought detection, host–parasitic plant relationships, floral thermogenesis, floral cooling, ice nucleation, seed vigour assessment and responses to abiotic and biotic stresses. Beyond illustrating individual physiological phenomena, the activities emphasise the unifying principle that infrared thermography does not simply measure temperature but provides an integrated window into plant physiology by revealing processes that would otherwise be invisible. At the same time, students become familiar with a research tool that is increasingly used in plant science, precision agriculture and crop phenotyping. As infrared cameras become more affordable and accessible, thermography represents a powerful and realistic approach to enriching inquiry-based learning and strengthening the connection between undergraduate teaching and contemporary plant research.

## 1. Introduction

Plant physiology encompasses numerous dynamic processes that are inherently difficult for students to observe directly. Processes such as transpiration, stomatal regulation, drought responses, graft union formation, plant–pathogen interactions and floral thermogenesis are typically inferred from indirect measurements or described using conceptual models. Consequently, students often find it challenging to connect theoretical concepts with observable plant behaviour.

Infrared thermography provides an effective way to overcome this limitation by providing spatially resolved, non-invasive measurements of surface temperature [1], Because plant temperature reflects the interaction between transpiration, stomatal conductance, plant water status and energy exchange with the environment, thermal images provide an integrated visual representation of physiological function. Rather than simply generating temperature maps, thermal imaging enables students to visualise temperature differences across plant tissues and investigate the physiological consequences of processes that are otherwise difficult to observe directly. The educational value of thermography therefore lies not simply in producing visual images, but in using temperature patterns to investigate the physiological mechanisms underlying plant function [1].

Until recently, the widespread use of infrared thermography in teaching was limited by the high cost of thermal cameras [2,3]. Recent technological advances have substantially reduced these costs, making portable handheld cameras—and even smartphone-based thermal sensors—more affordable for many teaching laboratories. Consequently, infrared thermography has become accessible to universities and, increasingly, to secondary education [3].

At the same time, infrared thermography has become an established research tool across many areas of plant science, with applications ranging from drought physiology and plant–pathogen interactions to graft compatibility, freezing events, seed quality assessment and floral thermogenesis [4].

Despite this growing research interest, comparatively little attention has been devoted to the educational applications of infrared thermography in plant physiology. Infrared thermography has been used in science education [1], but far more frequently in physics [5] and chemistry [6] than biology. In biology, previous educational reports have focused mainly on animal and human physiology, homeostasis or general biological demonstrations [1,3,7,8,9], whereas relatively few studies have specifically explored how thermal imaging can be used to teach core concepts in plant physiology [10]. Articles on the applications of thermal cameras in plant biology education have often targeted secondary school teachers [3,10,11], as well as primary school teachers [12]. While some previous work has interesting applications that could be used in university teaching, essentially no studies explore the use of thermographic cameras in teaching plant biology except in introductory biology courses, where they are used to illustrate thermogenic plants [13]. Recently, however, they have been used in an Agronomic Sciences degree to conduct comparative studies of well-watered and water-stressed plants [14].

Therefore, although infrared thermography has been used in science education and in selected university-level plant science courses, published resources specifically designed to integrate a broad range of plant physiological processes into inquiry-based undergraduate teaching remain limited. This article addresses this gap by bringing together ten adaptable activities that connect thermal observations with physiological questions, experimental reasoning and the interpretation of plant function in undergraduate plant physiology teaching. The activities can be adapted for courses in plant physiology, environmental plant physiology, applied plant physiology and plant protection. Rather than providing an exhaustive review of thermal imaging as a technique, we offer a conceptually integrated teaching resource focused on activities that instructors can adapt to their own courses.

Each activity uses a physiological question to guide students from predicting a thermal response to interpreting experimental observations. Students are encouraged to formulate hypotheses, interpret thermal patterns and relate temperature patterns to underlying physiological mechanisms, thereby linking theoretical knowledge with experimental evidence. In this way, the activities connect fundamental plant physiology with scientific reasoning, while introducing students to a research technique that is becoming increasingly prevalent in plant science and agricultural applications.

## 2. Fundamentals of Infrared Thermography

All objects with a temperature above absolute zero emit infrared radiation. The amount of radiation emitted depends on surface temperature and emissivity, allowing thermal cameras to estimate temperature without physical contact [15]. Infrared cameras detect radiation within the thermal infrared region (approximately 3–14 μm) and convert it into a false-colour image known as a thermogram, in which each pixel represents the temperature of a small area of the observed object [16]. Temperature differences within the scene therefore become immediately visible, enabling users to identify thermal patterns that would otherwise remain undetected.

In plants, these temperature differences frequently reflect underlying physiological processes because leaf temperature is strongly influenced by transpiration, stomatal conductance, plant water status and energy exchange with the surrounding environment. Therefore, thermograms provide indirect evidence of physiological function. However, their interpretation requires consideration of both plant physiology and the physical conditions under which the images were acquired.

### 2.1. Obtaining Reliable Thermograms in the Teaching Laboratory

Although infrared thermography is straightforward to use, obtaining meaningful thermal images requires careful control of the experimental conditions. For most teaching activities, the aim is to compare relative temperature differences between treatments rather than to obtain highly accurate absolute temperature measurements. Standardising the measurement conditions is therefore essential.

Several factors should be considered when acquiring thermograms. Environmental conditions such as solar radiation, ambient temperature, wind and relative humidity influence leaf temperature independently of plant physiology and should therefore be kept as constant as possible throughout the experiment. Whenever feasible, thermal images should be acquired under diffuse light or in shaded conditions to minimise uneven heating and thermal reflections caused by direct sunlight.

The distance between the camera and the plant should remain constant, and the target should occupy a sufficient number of pixels to ensure reliable temperature measurements. Plant surfaces generally have high emissivity, making them well suited to infrared thermography; however, reflective materials such as metal labels, wet surfaces and glossy backgrounds should be avoided because they may introduce measurement artefacts.

Students should interpret thermograms in the context of the experimental design rather than assume that every temperature difference represents a physiological response, since leaf temperature reflects the combined influence of transpiration, plant water status, energy balance and environmental conditions. This distinction provides an excellent opportunity to discuss the limitations of experimental measurements and the importance of controlling environmental variables in physiological investigations.

### 2.2. Educational Applications of Infrared Thermography in Plant Physiology

Before undertaking the proposed activities, students should have an understanding of the basic principles of infrared thermography and the plant physiology concepts relevant to each task, as specified in the corresponding section. The level of prior knowledge and the depth of analysis can be adjusted to the course and student level. The ten activities are presented below.

#### 2.2.1. Can Plants Cool Themselves? Visualising Transpiration and Stomatal Regulation

Infrared thermography provides a simple and intuitive way to visualise how stomatal aperture regulates transpiration and, consequently, leaf temperature through evaporative cooling (Figure 1).

Students with prior knowledge of plant anatomy and water relations should be able to relate stomatal conductance to transpiration and leaf temperature, predict thermal responses to contrasting environmental conditions, and interpret infrared images using physiological principles.

Well-watered plants can be compared under light and dark conditions, or with plants subjected to other treatments that reduce transpiration, such as water deficit or abscisic acid (ABA) application. These treatments should be treated as separate experimental comparisons rather than as interchangeable controls. Species with relatively large leaves, including *Tradescantia pallida*, *Tradescantia zebrina*, *Viola* spp. and *Petunia* spp., are suitable because they provide clearly resolved thermal images. *Tradescantia* species are particularly useful because the same material can also be used to observe stomatal structure under the microscope. For the light-versus-dark comparison illustrated in Figure 1A–D, eight independent 500 mL pots containing peat and *T. pallida* plants approximately 20–30 cm tall were assigned to each condition. The leaf temperature and room temperature were recorded for each plant, and ΔT was calculated using the same definition for all observations (Figure 1E).

Under otherwise comparable conditions, darkness may promote stomatal closure and reduce transpiration, whereas illumination may promote stomatal opening and evaporative cooling. The resulting leaf-temperature response is nevertheless influenced by irradiance, leaf energy balance, vapour-pressure deficit, air movement and plant water status. Therefore, the expected direction and magnitude of the temperature difference should be treated as a hypothesis to be tested under the actual measurement conditions, rather than as an invariant response.

This activity demonstrates that transpiration serves not only as the driving force for water transport but also as an effective mechanism for regulating leaf temperature. By comparing thermal images obtained under contrasting conditions, students discover that leaf temperature reflects not only ambient conditions but also stomatal behaviour and plant water status. More broadly, the experiment illustrates how infrared thermography provides a simple, rapid, non-destructive means of visualising physiological processes that are otherwise invisible.

This activity is suitable for undergraduate introductory biology and plant physiology programs in the section on water relations, providing the necessary context for the experiments.

#### 2.2.2. Can a Thermal Camera Tell Whether a Graft Has Healed? Monitoring Graft Union Formation

Infrared thermography enables students to visualise how the restoration of vascular continuity following grafting influences water transport, transpiration, and leaf temperature.

In grafting, usually the root part of a plant (rootstock) is combined with the aerial part of another plant (scion) to form a new plant. Students aware of the background of the grafting process should be able to explain why newly grafted scions experience water stress, interpret changes in leaf temperature during graft union formation, and relate thermal patterns to the progressive recovery of xylem functionality.

This experiment can be conducted using 20 one-month-old grafted tomato plants, for example (Figure 2A, [17]). Following grafting, students monitor the plants at regular intervals (e.g., 0, 2, 4 and 6 days after grafting—DAG) using an infrared camera. Students can then plot the temperature differences between individual plants and the room temperature through the experiment and calculate the relevant statistical parameters (Figure 2B).

Thermal images of the scion are compared over time and, whenever possible, with those of intact, non-grafted plants. Before analysing the thermograms, students predict how leaf temperature may change as the graft union develops and justify their predictions in terms of plant–water relations. If the same plants are measured repeatedly, the analysis should account for the repeated-measures design.

Immediately after grafting, the hydraulic connection between the rootstock and scion is disrupted. The scion may consequently experience water deficit, stomatal closure and reduced transpiration, which can reduce evaporative cooling and cause the leaf temperature to approach ambient temperature. As the graft union develops and hydraulic function is restored, water transport and transpiration may recover, leading to lower leaf temperatures under otherwise comparable conditions. The thermal response is therefore consistent with the recovery of evaporative cooling, but does not by itself demonstrate the restoration of xylem continuity.

This activity illustrates how infrared thermography may provide a rapid, non-destructive means of monitoring physiological changes associated with graft union development. Thermal imaging does not visualise the graft union itself and cannot independently confirm vascular reconnection. Rather, it provides an indirect indicator of changes in leaf temperature that may be associated with plant water status, stomatal regulation and evaporative cooling. Students should therefore interpret thermal observations alongside other evidence of graft success and consider alternative explanations for temperature changes and leaf energy balance, while introducing an increasingly important application of infrared thermography in horticultural research.

Therefore, this activity would be a good addition to an undergraduate program in applied plant physiology or plant production, particularly if it included a section on grafting.

#### 2.2.3. Can a Thermal Camera Detect Disease Before Symptoms Appear? Investigating Plant–Pathogen Interactions

Infrared thermography enables students to visualise how plant pathogens alter stomatal behaviour and how these physiological responses influence leaf temperature.

Students with a prerequisite knowledge of plant–pathogen interactions should be able to explain how pathogen infection affects stomatal conductance, interpret local changes in leaf temperature as indicators of altered transpiration, and appreciate the potential of thermal imaging for the early detection of plant diseases.

Students compare thermograms of healthy plants with those of plants infected by a foliar pathogen, such as *Pseudomonas syringae* pv. tomato DC3000 in tomato plants, approximately one week after inoculation (Figure 3), or analyse published thermal images when infected material is unavailable. Before examining the images, they predict whether infected tissues may appear warmer or cooler than healthy tissues and justify their hypotheses in terms of transpiration and stomatal behaviour. Appropriate mock-inoculated controls and biological replication are required for a quantitative comparison.

In many plant–pathogen interactions, stomatal closure is among the early plant responses, although the direction and magnitude of the response depend on the pathogen, infection stage and host response [18]. When stomatal conductance and transpiration decline, evaporative cooling may be reduced and infected tissues may become warmer than surrounding healthy areas. Such thermal changes have been reported before visible symptoms in some pathosystems [19,20,21], but this response is not universal.

This activity demonstrates that infrared thermography detects the physiological consequences of pathogen infection rather than the pathogen itself. The observed thermal patterns reflect changes in stomatal conductance and plant–water relations, allowing students to understand how physiological responses frequently precede visible disease symptoms. Students can discuss why stomatal closure is part of the plant–pathogen interaction, how different pathogens may elicit distinct thermal patterns, and why infrared imaging has become a valuable tool for the early detection and monitoring of plant diseases.

To stimulate inquiry, students can be asked: can plants develop a fever? In some thermal images, infected tissues appear warmer than surrounding tissues. However, this does not mean that plants develop fever through an animal-like thermoregulatory response. Local warming may instead result from reduced evaporative cooling, changes in energy balance or other infection-associated processes. The thermal response can vary with the pathogen, infection stage and host response; therefore, thermography alone can diagnose a disease, though it should not be treated as a diagnostic test.

This activity fits well with an undergraduate program in environmental plant physiology, particularly in the section on biotic stresses of plants, but it could also be used in a crop production program.

#### 2.2.4. Can We Detect Drought Before Plants Wilt? Optimizing Irrigation Scheduling

Infrared thermography enables students to visualise how water deficit alters stomatal behaviour, leaf temperature and plant water status, allowing drought stress to be detected before visible symptoms appear.

Should irrigation begin when plants first become warmer, or only after visible wilting develops? Students explain why water-stressed plants become warmer before visible wilting occurs, interpret thermal images as indicators of plant water status, and appreciate the role of infrared thermography in precision irrigation.

Students who have completed activity 2.2.1 and are familiar with water relations will be able to compare well-watered control plants with identical plants that have been subjected to progressive water deprivation (e.g., 0, 2, 4, 6, 8 and 10 days without irrigation), with the sampling interval adjusted to the species, pot size and environmental conditions. One-month-old tomato plants (Figure 4A) or young maize plants (Figure 4B,C) are suitable for this experiment. At each sampling time, thermal images are recorded and leaf temperatures are compared among treatments. They identify the earliest stage at which thermal differences become detectable and discuss when irrigation should be applied to prevent irreversible physiological damage.

Thermal infrared imaging is especially useful for identifying progressive water stress, as physiological changes occur before visible symptoms appear. As soil water availability declines, stomata progressively close to reduce water loss through transpiration. The resulting decrease in evaporative cooling causes leaf temperature to increase, often several days before plants exhibit visible wilting symptoms. Consequently, thermal imaging reveals early physiological responses to drought that would not be apparent from visual inspection alone. However, the magnitude and timing of the response depend on environmental conditions, the species and the characteristics of the plant and the measurement procedure. Therefore, the first detectable thermal difference should be interpreted as an experimental observation rather than an automatically validated irrigation threshold.

This activity introduces one of the most important agricultural applications of infrared thermography. Thermal images acquired from handheld cameras, unmanned aerial vehicles (UAVs) or other remote-sensing platforms are increasingly used to assess crop water status and calculate thermal indices such as the Crop Water Stress Index (CWSI), providing growers with real-time information for irrigation scheduling [22,23,24,25]. This technique has been shown to detect drought stress in living wall panels earlier than visual assessment can, and it is most useful when water stress develops gradually [26]. Similar approaches have been successfully applied to fruit crops and other agricultural systems, while thermal phenotyping is increasingly employed to identify cultivars with enhanced tolerance to drought, salinity and low-temperature stress [23,24].

By linking laboratory observations with these real-world applications, students gain insight into how plant physiological principles underpin modern precision agriculture.

This activity is suitable for both an applied plant physiology program and a crop production program, specifically in the section devoted to watering.

#### 2.2.5. Why Is Mistletoe Cooler than Its Host, Whereas Dodder Usually Is Not? Investigating Host–Parasitic Plant Interactions

Infrared thermography enables students to explore how transpiration, stomatal conductance, and host–parasite water relations shape plant temperature.

Students are first presented with a simple but intriguing question: “Two parasitic plants depend on their hosts for water and nutrients. Why does one appear several degrees cooler than its host, whereas the other is almost thermally indistinguishable?” At first glance, students may expect parasitic plants to share similar thermal characteristics because they depend on their hosts for water and nutrients. Instead, thermal imaging reveals that parasite temperature is determined by how water is acquired, transported and lost, rather than by the parasitic lifestyle itself.

Students with a preliminary background in plant–water relations and host–parasite interactions should be able to explain why different parasitic plants exhibit distinct thermal signatures, relate plant temperature to transpiration and evaporative cooling, and recognise that thermal patterns reflect physiological function rather than parasitic lifestyle alone.

Students obtain thermal images of host plants infected by different parasitic species, such as mistletoes and dodders, or alternatively analyse published thermograms [27] when living material is unavailable. When possible, dodder seeds can be collected from alfalfa infestations and placed on a P60 Petri dish lined with two moistened filter paper discs to germinate in a growth chamber in the dark at 23 ± 1 °C. Once germinated, the dodder seedlings can be used to infest a batch of three- to four-week-old tomato or mung bean plants in order to observe the progression of the infestation [28]. Mistletoes are more difficult to culture and are best photographed in their natural habitat.

Before interpreting the images, they predict whether each parasite should appear warmer or cooler than its host and justify their hypotheses in terms of transpiration, stomatal conductance, leaf morphology, water acquisition strategy and vascular connections.

Mistletoes (e.g., *Viscum album* or *Phoradendron* spp.) typically appear cooler than the surrounding host canopy because they maintain high stomatal conductance and transpiration rates, generating substantial evaporative cooling. Their low water potential sustains a continuous flow of water from the host xylem, and the thermal difference becomes particularly evident under conditions of high irradiance, elevated air temperature and low relative humidity. In contrast, dodders (*Cuscuta* spp.) usually exhibit temperatures similar to those of the host stem. Although they establish functional connections with both xylem and phloem, their small transpiring surface, limited stomatal development, and low transpiration rates result in little evaporative cooling and, consequently, minimal thermal contrast with the host.

This activity demonstrates that plant temperature is governed by physiological processes rather than by ecological lifestyle. Students therefore recognise that infrared thermography does not distinguish parasitic plants from non-parasitic plants; instead, it reveals how different physiological strategies influence plant energy balance. These contrasting strategies provide an excellent opportunity to discuss plant–water relations, host–parasite interactions, and the relationship between plant structure and physiological function. Similar thermal patterns have been reported in naturally infected eucalyptus trees, where mistletoe foliage remained consistently cooler than the host canopy, particularly during periods of high solar radiation [27].

This activity is well suited to an undergraduate plant physiology program, and even more so to an environmental plant physiology program, in the section devoted to plant–plant interactions. It is also suitable for applied plant physiology programs and even crop production programs, in sections dedicated to crop losses due to parasitic plants.

#### 2.2.6. Can Plants Produce Heat? Exploring Floral Thermogenesis

Infrared thermography enables students to visualise one of the most remarkable examples of plant metabolism: the active production of heat by the flowers of thermogenic species.

Students with a basic understanding of thermogenic plants and in flowering would be able to explain the physiological basis of floral thermogenesis, interpret thermal images of thermogenic flowers, and discuss the ecological significance of heat production in plant–pollinator interactions.

Students obtain thermal images of thermogenic flowers, either from living specimens, when available, or alternatively by analysing published thermograms when fresh material is unavailable. Suitable examples include the inflorescences of *Arum* spp. and the flowers of the sacred lotus (*Nelumbo nucifera*) and water lilies (*Nymphaea* spp.) [8,9,29,30]. Before examining the images, they are asked whether flowers should have the same temperature as the surrounding air and to propose possible mechanisms by which plants could increase the temperature of their reproductive organs.

Unlike most flowering plants, thermogenic species maintain floral structures several degrees warmer than the surrounding air. Thermal images reveal warm floral organs that contrast with adjacent leaves and the surrounding environment, providing visual evidence consistent with metabolic heat production. Floral thermogenesis is a rare but remarkable adaptation that has evolved independently in several plant lineages. Heat is generated through intense respiratory activity, largely involving the alternative oxidase pathway, and may enhance the volatilisation of floral scents, increase pollinator attraction by providing a thermal reward, or protect sensitive reproductive tissues under cool environmental conditions [29,30]. The relative importance of these functions varies among species and should be discussed in relation to the available evidence.

Because conventional thermometers provide only point measurements, infrared thermography offers a rapid, non-invasive method for visualising both the spatial distribution and temporal dynamics of heat production.

This activity challenges one of the most common misconceptions among students: that plants simply adopt the temperature of their surroundings and are incapable of generating heat. The striking thermal contrast displayed by thermogenic flowers provides compelling evidence that plant temperature is not solely determined by the environment but can also be actively regulated through metabolism [31].

This activity is suitable for undergraduate programs in environmental plant physiology, in the section devoted to plant–animal interactions. It could also be used in general plant physiology programs if a section is devoted to flowering and thermogenic plants.

#### 2.2.7. Can Flowers Stay Cooler than Leaves? Exploring Solar Heating and Floral Cooling

Infrared thermography enables students to investigate how differences in transpiration and energy balance influence the temperature of floral and vegetative organs in some plant species.

Students are asked: if flowers receive more sunlight than leaves, shouldn’t they always be warmer? Students interpret why flowers and leaves of the same plant may differ in temperature, relate floral cooling to transpiration and energy balance, and discuss the ecological significance of floral temperature regulation.

Students with a general understanding of transpiration, flowering and energy balance can use an infrared camera to compare the surface temperature of flowers and leaves from the same plant at different times of day. In this activity, direct sunlight is an experimental variable rather than a condition to be avoided; measurements should therefore be accompanied by records of irradiance, ambient temperature, relative humidity, wind and camera orientation. Species with conspicuous flowers, such as summer-flowering Asteraceae, are particularly suitable [32]. Before collecting the thermal images, students are asked to predict which organs will become warmer under solar radiation and justify their hypotheses based on differences in transpiration, morphology and exposure to sunlight.

Under hot environmental conditions, some non-thermogenic flowers and inflorescences can maintain surface temperatures substantially below ambient air temperature [32,33]. These differences reflect the combined effects of radiative energy exchange, convective heat transfer, evaporative cooling and floral traits such as morphology, orientation and optical properties [34,35].

In Mediterranean summer-blooming Asteraceae, this cooling may benefit both reproductive tissues, by reducing heat stress, and flower-visiting insects, by providing relatively cool microhabitats during periods of high ambient temperature [32]. The thermal contrast generally becomes more pronounced under periods of intense solar irradiance.

Moreover, floral temperature is not necessarily distributed uniformly across the flower surface, and distinct floral structures may exhibit contrasting temperatures, contributing to the formation of distinct floral microclimates [36]. Depending on species and environmental conditions, some floral structures may also become warmer than the surrounding air as a result of absorbed solar radiation, without requiring metabolic heat production [34,35,36]. Rather than discouraging activity, these observations provide a valuable opportunity to explore the complex relationships between floral structure, energy balance and the environment. Solar irradiance can increase floral temperatures and, under some conditions, accentuate thermal contrasts within flowers, although the magnitude and spatial distribution of these contrasts depend on floral traits and environmental conditions.

By comparing flowers and leaves from the same individual, students realise that the temperature of plant organs is actively regulated by physiological and structural traits rather than determined solely by the surrounding environment.

Lower floral temperatures may help to prevent overheating of reproductive tissues and, in some species, provide a more favourable microclimate for visiting pollinators, potentially affecting pollinator activity and reproductive success [32]. These potential ecological benefits should be distinguished from the direct thermal observations, which demonstrate temperature differences but do not by themselves establish an effect on pollinator behaviour or reproductive success [35]. The activity also encourages discussion of the multiple factors influencing organ temperature, including transpiration, flower architecture and geometry (i.e., petal size and thickness, and corolla shape), petal colour and its influence on radiation absorption, orientation relative to the Sun, wind exposure and radiative energy balance.

When considered alongside the previous activity on floral thermogenesis, this activity reinforces an important concept: flowers are not thermally passive organs. Some species actively generate heat, whereas others maintain relatively cool flowers through the combined effects of transpiration and energy balance. Together, these contrasting strategies illustrate how temperature regulation contributes to reproductive function and plant–pollinator interactions.

As with the previous activity, this activity is suitable for undergraduate courses in environmental plant physiology, particularly in sections devoted to plant–animal interactions. It could also be used in general plant physiology courses, particularly in sections devoted to pollination, including entomophilous pollination.

#### 2.2.8. Can Freezing Make a Leaf Warmer? Visualising Ice Nucleation and Ice Propagation in Plants

Infrared thermography enables students to illustrate how ice nucleates and spreads through plant tissues, revealing freezing events that are invisible to the naked eye.

Students explain why the formation of ice is accompanied by the release of latent heat, interpret thermal images showing ice nucleation and propagation, and discuss the significance of freezing dynamics for plant survival and crop protection.

Students with knowledge of thermodynamics and plant responses to freezing can observe thermal videos of detached leaves or shoots during controlled freezing experiments or analyse published thermograms when specialised equipment is unavailable. For example, leaves or whole plants of *Phaseolus vulgaris* can be placed in a suitable thermal chamber and equilibrated initially at approximately 1 °C for 30–60 min [37,38]. The chamber temperature is then lowered progressively while thermographic images or video are recorded at a frequency sufficient to resolve the onset and propagation of freezing.

Before examining the recordings, students are asked to predict whether freezing should make plant tissues warmer or colder and to justify their answer using basic principles of thermodynamics.

Although freezing ultimately leads to lower tissue temperatures, the initial formation of ice crystals is accompanied by the release of latent heat of fusion. As a result, thermal images reveal a brief and localised increase in temperature at the site of ice nucleation, followed by the rapid propagation of a thermal front as freezing spreads through the tissue. This transient warming provides a direct visualisation of an otherwise invisible physical process, namely the progression of freezing [37]. Importantly, infrared thermography does not visualise the ice crystals themselves; rather, it detects the transient release of latent heat that accompanies ice formation.

This activity illustrates how infrared thermography offers a unique, non-invasive method for studying freezing dynamics in living plants, with high spatial and temporal resolution. These observations help students understand that freezing is a dynamic physical process rather than an instantaneous event and illustrate the close relationship between plant physiology and thermodynamics. Beyond its educational value, thermal imaging has become an important research tool for investigating frost resistance, ice barriers, and freezing tolerance in crops and wild species [38]. Because frost damage represents one of the leading causes of agricultural losses worldwide, understanding how and where ice forms within plant tissues has considerable agronomic significance.

This activity is of particular interest in the context of an environmental plant physiology program, specifically in the section devoted to the effects of freezing on plants.

#### 2.2.9. Can a Thermal Camera Predict Whether a Seed Will Germinate? Assessing Seed Vigour Through Active Infrared Thermography

Active infrared thermography enables students to investigate how seed ageing alters the thermal properties of seeds and how these changes can be exploited to assess seed vigour rapidly and non-destructively.

Students who are familiar with the physiology of seed germination can be asked: “Should vigorous and aged seeds cool at the same rate?” Students explain how seed deterioration affects thermal behaviour, distinguish between passive and active infrared thermography, and interpret cooling curves as indicators of seed quality and germination potential.

As a suggested activity, students compare seeds of contrasting vigour, such as freshly harvested and artificially aged seeds. Batches of seeds can be selected (e.g., 50 seeds per treatment), using a species for which the active-thermography procedure has been validated [39,40]. The sequence and conditions of imbibition and artificial ageing must follow the cited validated method; these steps should not be combined or reordered without verification. After the treatment, seed temperature is equilibrated under controlled conditions and a brief, standardised heat pulse is applied using a suitable heat source. Thermal images are recorded during cooling and the thermal decay of individual seeds is analysed. The heat pulse must be calibrated for each species and seed lot, and its effect on subsequent germination must be tested.

Before analysing the results, students are asked to predict whether vigorous and aged seeds should cool at the same rate and to justify their hypotheses based on changes associated with seed ageing.

Although seeds may reach similar temperatures after heating, they can dissipate heat at different rates depending on their physical and physiological condition. Seed ageing can alter tissue structure, water distribution and thermal properties, leading to measurable differences in thermal decay. Differences in thermal relaxation have been associated with seed vigour and germination potential in particular, experimental systems [40]. The outcome may depend on species, hydration status, seed size and thermal treatment. Accordingly, the heating procedure must be adapted to the seed species and validated to ensure that it does not compromise viability.

Unlike the previous activities, which rely on passive thermal imaging of naturally occurring temperature differences, this experiment introduces active infrared thermography, in which a controlled heat stimulus enhances otherwise imperceptible thermal contrasts [41]. The camera records how seeds absorb, store and dissipate externally supplied heat; it does not detect metabolic heat production. Depending on the method and analysis, the results may be expressed using parameters describing thermal decay or thermal transport. The activity also provides an opportunity to discuss the physiological consequences of seed ageing, including membrane deterioration, reduced metabolic activity and declining germination capacity [40]. This non-destructive approach has attracted increasing interest in seed technology because it enables rapid assessment of seed quality without compromising viability [39].

This activity is suitable for inclusion in a plant physiology program in the section devoted to seed physiology. More specifically, this activity fits well into applied plant physiology and crop physiology programs in the section devoted to seed technologies, as well as Master’s programs specializing in plant propagation.

#### 2.2.10. Can Thermal Imaging Detect Hidden Plant Stresses? Monitoring Abiotic and Biotic Stress Responses

Infrared thermography enables students to investigate how diverse abiotic and biotic stresses alter plant physiological processes and how these changes can be detected before visible symptoms develop [42].

Students with background knowledge of plant stress responses should be able to explain how different abiotic stresses influence stomatal conductance and plant–water relations, interpret thermograms as indicators of physiological stress, and appreciate the value of infrared thermography for the early detection of plant dysfunction.

Students compare thermograms of healthy plants with those subjected to selected abiotic stresses, such as salinity, flooding or nutrient deficiency. These should be treated as separate case studies, each with an appropriate control, a defined stress treatment and a physiological hypothesis. The approach is similar to that described for plant–pathogen interactions (Section 2.2.3) and water deficit (Section 2.2.4). Whenever possible, images are acquired before visible symptoms develop. Students predict how each selected stress may affect leaf temperature and discuss the mechanisms that could explain the observed patterns.

Many abiotic stresses can alter stomatal conductance, transpiration and leaf energy balance, but they do not all produce the same thermal response. The magnitude, timing and spatial distribution of temperature changes depend on the type, severity and duration of stress, the species and its physiological response, and the prevailing environmental conditions [4]. Thermograms may reveal physiological alterations before visible symptoms in some experimental systems, but this should be established for each stress–species combination rather than assumed.

This approach is also applicable to biotic stresses. For example, leaves of sunflowers inoculated with the parasitic weed *Orobanche cumana* were warmer than the control leaves [43]. Similarly, increases in canopy temperature enabled the early detection of late wilt disease in maize, which is caused by the soil-borne fungus *Harpophora maydis* [44], as well as the identification of olive trees infected by *Verticillium dahliae* in field conditions [45].

As the final activity of this series, this exercise brings together many of the concepts introduced throughout the previous sections. Rather than diagnosing a particular stress type, infrared thermography reveals alterations in plant function, allowing students to recognise that diverse environmental constraints frequently converge on common physiological pathways, particularly those involving stomatal regulation, water relations and energy balance. This integrative perspective underpins many contemporary applications of thermal imaging in crop monitoring, precision agriculture and plant phenotyping [23,24], while reinforcing the central message of this article: infrared thermography does not simply measure temperature; it provides a window into plant physiology.

This activity would be a good addition to an environmental plant physiology program, particularly in the section devoted to abiotic and biotic stresses.

## 3. Educational Perspectives

The activities presented in this article are grounded in an inquiry-based learning approach. A distinctive feature of all the activities is that they begin with a provocative question rather than with an explanation. Students with a background in different areas of plant physiology, depending on the activity, are first invited to predict the expected thermal response of the plant, then acquire or analyse thermograms, and finally interpret the observed temperature patterns in terms of the underlying physiological processes. This sequence encourages students to formulate hypotheses, test their predictions against experimental evidence and refine their understanding through discussion.

Many of the proposed activities deliberately challenge intuitive assumptions. For example, students are asked whether plants can cool themselves, whether flowers can produce heat, or whether freezing can transiently warm a leaf. Such questions create cognitive conflict that stimulates conceptual change and encourages students to integrate knowledge from plant–water relations, energy balance, plant–environment interactions and metabolism.

Thermograms provide an immediate visual representation of temperature patterns that may help students make connections that are otherwise difficult to observe directly. As a result, they facilitate conceptual understanding by linking theoretical principles with experimental observations. The activities are designed to create opportunities for students to interpret plant temperature as an integrated physiological response rather than as an isolated physical variable. They may also support the development of experimental design, data interpretation and scientific reasoning. However, these potential educational benefits have not been systematically assessed across the ten activities.

The proposed activities are based on well-established concepts in plant physiology and draw on findings from different areas of the discipline. Nevertheless, infrared thermography provides evidence of thermal patterns associated with plant function rather than revealing the underlying processes directly. The potential educational benefits of the activities remain to be evaluated systematically. Their implementation in defined undergraduate courses, followed by appropriate assessment of student learning and feedback, is therefore an important direction for future work.

Temperature should therefore be interpreted as an indirect indicator of plant function whose meaning depends on an understanding of the underlying physiology. This limitation is, in itself, an important educational opportunity, as it encourages students to distinguish between observations and their biological interpretation.

## 4. Conclusions

Infrared thermography offers a powerful and accessible means of making plant physiology visible. By transforming temperature into a physiological signal, thermal imaging allows students to observe processes that are otherwise inferred only indirectly, thereby strengthening the connection between theoretical concepts and experimental evidence.

The ten inquiry-based activities presented here demonstrate that infrared thermography can support teaching across a wide range of plant physiology topics, including transpiration, graft union formation, plant–pathogen interactions, drought stress, host–parasite relationships, floral temperature regulation, freezing, seed quality and abiotic stress responses. Although these phenomena appear unrelated, they all illustrate the same underlying principle: plant temperature is an integrated physiological trait reflecting the interaction between metabolism, water relations and the environment.

Beyond enriching practical teaching, infrared thermography familiarises students with a research tool that is increasingly important in plant science, precision agriculture and plant phenotyping. As portable thermal cameras become more affordable, their incorporation into undergraduate teaching laboratories represents a potentially valuable strategy for promoting active learning and inquiry-based education while strengthening links between teaching, research and professional practice.

Ultimately, the educational value of infrared thermography lies not in the technology itself, but in its ability to reveal the physiological consequences of plant function. Students do not simply learn to measure temperature; they learn to interpret temperature as evidence of how plants work.

## Figures and Tables

**Figure 1:**
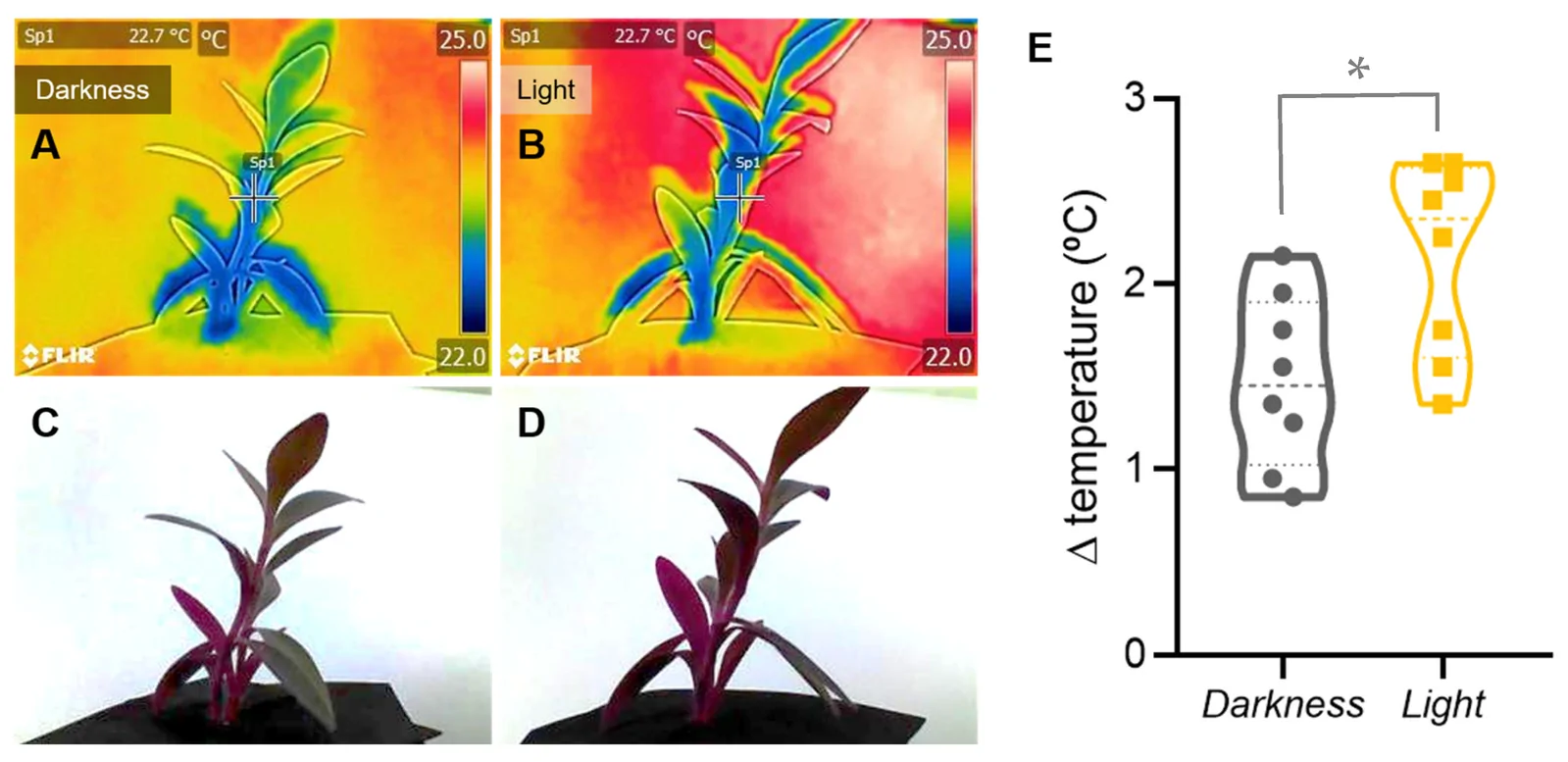
Representative *Tradescantia pallida* plants maintained in darkness (**A**,**C**) and in light (**B**,**D**). (**A**,**B**) are thermographic images, while (**C**,**D**) are the corresponding visible-light images. The temperature scale to the right of (**A**,**B**) ranges from 22.0 °C to 25.0 °C. In light, lower temperatures were observed across the aerial parts of plants, whereas in darkness, lower-temperature regions were restricted to the basal parts of plants. Images were captured using a FLIR C3 thermographic camera. (**E**) Violin plot showing the temperature difference between room temperature minus the temperature of *Tradescantia* plants (ambient T–plant T) under darkness and light conditions (*n* = 8 per treatment). The *t*-test indicates a significant difference between the two conditions, as shown by the asterisk (*p*-value = 0.018). Emissivity was set to 0.95. Images were captured between 12:00 and 14:00 h, with the camera positioned perpendicular to the plant at a distance of 30 cm. The rainbow palette was used for display. Quantitative measurements were obtained from the radiometric thermal files using FLIR Tools.

**Figure 2:**
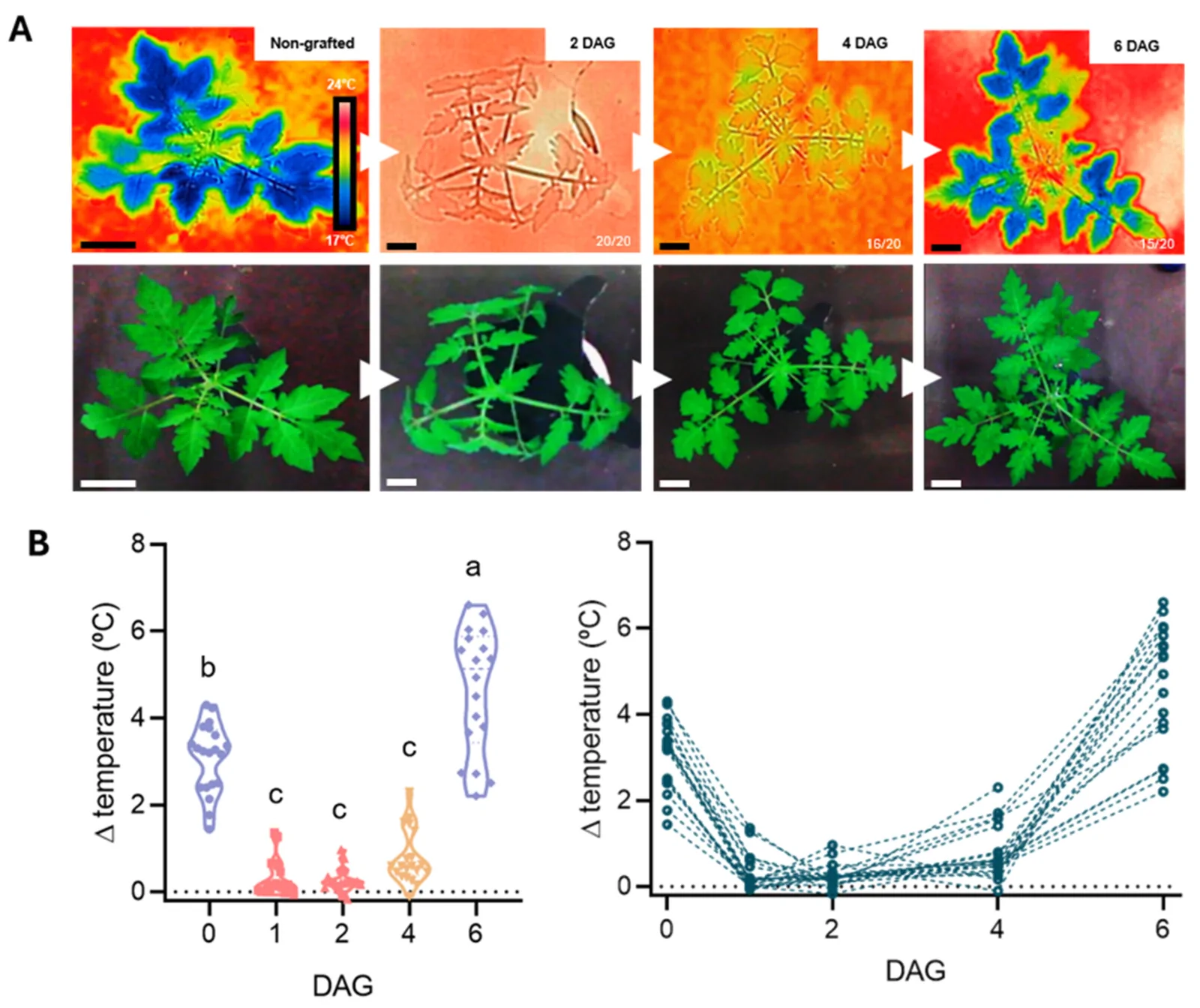
(**A**) Top: infrared thermograms showing representative tomato plants at 0 (non-grafted), 2, 4 and 6 days after grafting (DAG). The temperature scale is shown beside the first thermogram. Images were obtained using a FLIR C3 camera positioned vertically 30 cm above each plant, with emissivity set to 0.95. Plants were removed from the healing chamber and placed on a laboratory workbench close to the culture chamber. Images were captured at the same time of day under controlled conditions (22 ± 1.5 °C; 41% ± 2% relative humidity), with no detectable air flow. Temperature was measured from three points on the apical leaflets of the three most recently developed leaves of each plant and from three points on the room-temperature reference surface; each measurement point was a circle with a 5-pixel radius. The difference between ambient and leaf temperature (ambient T–leaf T) was used as an indirect thermal indicator associated with evaporative cooling, not as a direct measurement of transpiration. The X/20 label indicates the number of plants, out of 20, sharing the phenotype shown. Bottom: the same plants under visible light. Scale bars: 5 cm. (**B**) Left side: violin plots showing the temperature difference over the grafting period (*n* = 20). Different letters indicate significant differences at the *p*-value ≤ 0.05 level after repeated measures ANOVA analysis of variance followed by Tukey’s test. Right side: variation in inferred transpiration of the 20 individual plants as shown before.

**Figure 3:**
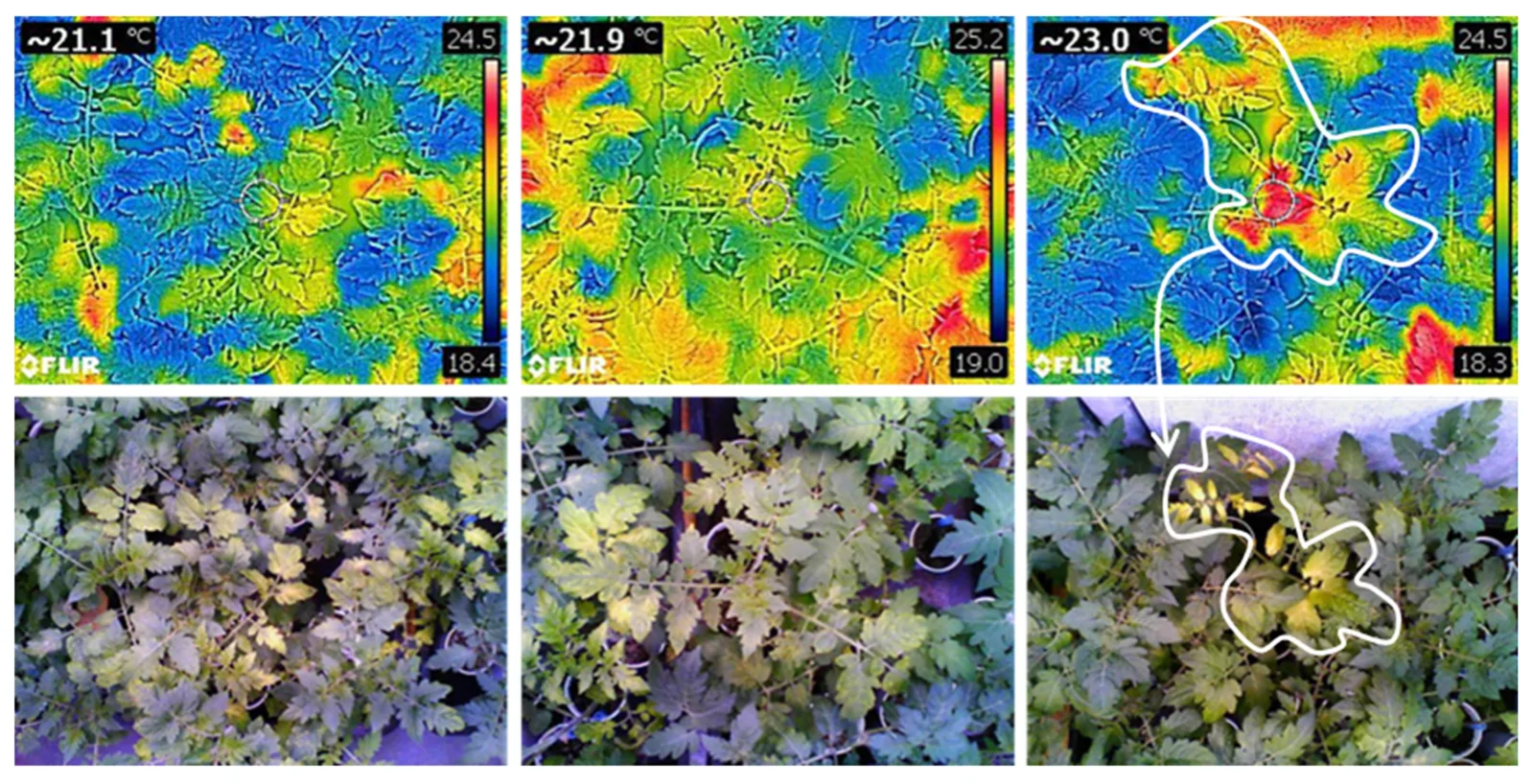
Top views of representative five-week-old tomato plants, one week after inoculation with *Pseudomonas syringae* pv. tomato DC3000. Thermograms are shown above the corresponding visible-light images. The temperature scale is shown beside the upper thermograms. The mean temperatures of the infected plants ranged from 21.1 to 23.0 °C. The warmer regions, marked by the white line in the top right image, correspond to the areas showing visible symptoms in the bottom right image. Images were captured using a FLIR C3 thermographic camera.

**Figure 4:**
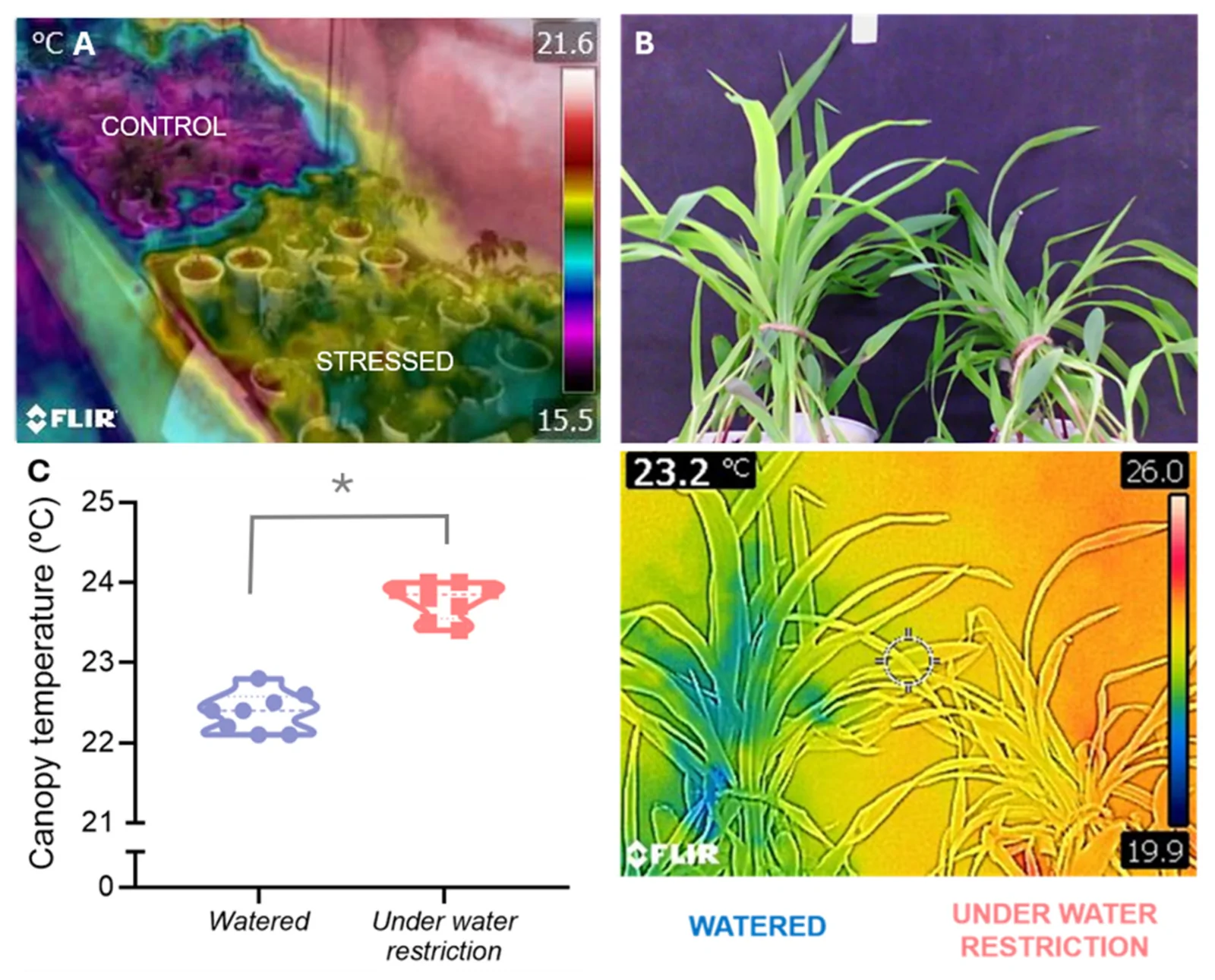
(**A**) Tomato plants (five-weeks old), with the control plants at the upper left and the unwatered plants at the lower right. The temperature scale ranges from white–red (high temperatures) to violet–black (low temperatures). (**B**) Young maize plants (three-weeks old), with watered plants on the left and water-restricted plants on the right. The upper image is a visible-light photograph, and the lower image is the corresponding thermogram. The temperature scale of the lower-right thermogram ranges from white–red (high temperatures) to blue–black (low temperatures) is shown on the right of the bottom right image. Images were captured using a FLIR C3 thermographic camera. (**C**) Canopy temperature quantification of randomized sampling areas of B thermographic images. The *t*-test indicates a significant difference between the two conditions, as shown by the asterisk (*p*-value: <0.001).

## Data Availability

The original contributions presented in this study are included in the article. Further inquiries can be directed to the corresponding authors.
